# Incorporating Milk Protein Isolate into an Energy-Restricted Western-Style Eating Pattern Augments Improvements in Blood Pressure and Triglycerides, but Not Body Composition Changes in Adults Classified as Overweight or Obese: A Randomized Controlled Trial

**DOI:** 10.3390/nu12030851

**Published:** 2020-03-22

**Authors:** Joshua L. Hudson, Jing Zhou, Jung Eun Kim, Wayne W. Campbell

**Affiliations:** 1 Department of Nutrition Science, Purdue University, West Lafayette, IN 47907, USA; JLHudson@uams.edu (J.L.H.); Hattie517844@gmail.com (J.Z.); fstkje@nus.edu.sg (J.E.K.); 2 University of Arkansas for Medical Sciences, Arkansas Children’s Nutrition Center Little Rock, AR 72202, USA; 3 Department of Food Science and Technology, University of Singapore, Singapore 119077, Singapore

**Keywords:** weight loss, health, dietary protein, muscle mass

## Abstract

Unhealthy Western-style eating patterns (WSEP) predominate, adversely affecting health. Resistance to improving dietary patterns prompts interest to incorporate a potentially health-promoting ingredient into typical WSEP foods and beverages. We assessed the effect of incorporating isocalorically matched carbohydrates versus milk protein isolate (MPI) into a WSEP on weight loss-induced changes in cardiometabolic health and body composition. In a randomized, double-blind, parallel-design study, 44 participants (age 52 ± 1 years, body mass index (BMI) 31.4 ± 0.5 kg/m^2^, mean ± standard error) consumed a weight maintenance WSEP (0.8 g total protein/kg/day) for 3 weeks (baseline). After, participants consumed an energy-restricted (750 kcal/day below estimated requirement) WSEP for 16 weeks, randomly assigned to contain either an additional 0.7 g carbohydrate/kg/d (CON: *n* = 23, 0.8 g total protein/kg/day) or 0.7 g protein/kg/d from MPI (MPI: n = 21, 1.5 g total protein/kg/day) incorporated into foods and beverages. Compared to CON, the MPI favored reductions in average 24 h and sleeping systolic and diastolic blood pressures (BP), waking hours systolic BP, and fasting plasma triglyceride concentrations. Reductions in fasting plasma insulin, glucose, total cholesterol, and low-density lipoprotein cholesterol concentrations were not different between groups. Among all participants, whole body mass, lean mass, fat mass, and thigh muscle area, each decreased over time. For adults finding it difficult to deviate from a WSEP, replacing a portion of their carbohydrate with foods and beverages containing MPI may be an effective dietary strategy to reduce BP after weight loss.

## 1. Introduction

Consuming a Western-style eating pattern (WSEP) ostensibly promotes positive energy balance, excess weight gain, and the development of metabolic syndrome among adults [1]. This is evident at a population level, where the prevalence of obesity has risen over the past 25 years while adherence to the Dietary Guidelines for Americans, which promotes consuming “healthy” eating patterns, has remained low [2]. Western-style eating patterns are characterized by high saturated fat, high added sugar, and low dairy intakes [1]. Accordingly, most Americans are not meeting the dairy recommendations [3]. Dairy consumption is inversely associated with metabolic syndrome prevalence and risks [4,5,6,7], while stronger evidence from several randomized controlled trials demonstrates that consuming dairy-based proteins may improve cardiometabolic health profile and body composition during periods of energy restriction [8,9]. The rising prevalence of overweight and obesity and slow progress towards adopting healthy eating patterns amongst the general population prompts interest in identifying new dietary interventions that include dairy-based proteins to promote health.

Consuming more dairy foods is scientifically plausible but may be practically challenging. In general, people are resistant to altering their habitual dietary patterns. This is apparent by low Healthy Eating Index (HEI) scores, a measure of how food choices align with the Dietary Guidelines for Americans, despite recommendations to consume a healthy diet [2]. Adherence diminishes over time even when research participants are prescriptively counseled to adopt a healthy eating pattern [10,11]. Milk protein isolate is made from fresh skim milk and retains most of the components of milk, including the proteins, peptides, and minerals, while most of the lactose is extracted. Previous research has added whole dairy foods [12,13] or single dairy proteins [14,15] to diets as a means of increasing dairy intake. However, since a WSEP remains a prominent dietary pattern and efforts to alter habitual food intakes are largely unsustainable, incorporating milk protein isolate into foods and beverages that conform to a WSEP may be one viable approach to potentially improve cardiometabolic health risk factors without altering habitual eating patterns. This strategy may be particularly effective for adults seeking to lose weight but who find it difficult to adopt healthier eating patterns.

Purposeful weight loss achieved by moderate dietary energy restriction is well recognized to effectively improve multifarious cardiovascular disease and type 2 diabetes mellitus risk factors in adults classified as overweight or obese [16]. Dietary strategies to promote or augment these improvements are sought, with higher dairy intake a plausible candidate [4,5,6,7,8,9].

The purpose of this study was to assess the effects of consuming an energy-restricted diet conforming to a WSEP with either an isocaloric carbohydrate control (maltodextrin) or milk protein isolate incorporated into foods and beverages on changes in both cardiometabolic health risk factors and body composition (whole body, fat, and lean mass), in middle-aged adults who are classified as overweight or obese. We hypothesized that compared with consuming foods and beverages containing carbohydrate, consuming foods and beverages containing milk protein isolate would promote greater improvements in blood pressure and plasma triglyceride concentrations and greater fat mass loss, and less lean mass loss.

## 2. Materials and Methods

### 2.1. Participants

Adults clinically classified as either overweight or obese were recruited from the Greater Lafayette, IN, community, using flyers posted to community boards. This is a population of adults where the effects of weight loss can be safely and ethically studied. Furthermore, over 70% of Americans are either overweight or obese, which is related with a host of morbidities such as Type II diabetes, stroke, and cancer [16]. Targeted weight loss interventions that promote changes in health among this population are of scientific interest. Inclusion criteria were: age 35–65 years, body mass index (BMI) 25–38 kg/m^2^, weight stable (±3 kg during previous 3 months), nonsmoking status, not acutely ill, not diabetic, not pregnant or lactating, not engaged in a structured exercise or weight loss program for ≥3 months prior to enrollment, lactose tolerant, natural waist circumference ≥ 102 cm for men and ≥88 cm for women, fasting serum glucose < 110 mg/dL, systolic and diastolic blood pressures < 140/90 mm Hg, serum total cholesterol < 260 mg/dL, low-density lipoprotein-cholesterol < 160 mg/dL, triglycerides < 400 mg/dL, and clinically normal serum albumin and pre-albumin concentrations.

### 2.2. Experimental Design 

This 20-week randomized, parallel, placebo-controlled, double-blind study included a 1-week pre-study measurement period, a 3-week baseline period, and a 16-week energy-restriction intervention period. During the pre-study week, participants’ habitual, self-chosen, diets were documented using food records filled out by the participants for two weekdays and one weekend day and analyzed by a registered dietitian using the Nutrition Data System for Research software (Version 2014; Nutrition Coordinating Center, University of Minnesota). During the 3-week baseline period, participants consumed a prescribed WSEP designed to provide their estimated energy requirement. At the pre-study week and at baseline week 3, body weight and cardiometabolic health risk factors were assessed. Body composition was assessed at baseline week 3. Participants were then randomized and assigned to one of two groups that consumed the same WSEP for 16 weeks with a 750 kcal/day energy deficit and containing selected foods and beverages with either carbohydrate (control: CON, *n* = 23) or milk protein isolate (MPI, *n* = 21) incorporated. Post-intervention testing was completed during intervention week 16. All participants were instructed to maintain their habitual types and levels of physical activities throughout the study. This study was conducted according to the 2013 Declaration of Helsinki guidelines. All procedures were approved by the Purdue University Biomedical Institutional Review Board and participants received a monetary stipend upon completion. This study is registered at clinicaltrials.gov as NCT01692860.

### 2.3. Dietary Intervention

Each participant’s total energy requirement was estimated using the Institute of Medicine sex-specific equations for adults who are overweight or obese with a low physical activity level [17]. During the 3-week weight-maintenance baseline period, each participant was prescribed a WSEP designed to meet their estimated energy requirement that contained 0.8 g protein/kg/day (Table 1). The non-protein energy ratio was 65% carbohydrate and 35% fat. Each participant was provided with approximately one-third of their estimated energy needs from select foods and beverages from our metabolic kitchen (a food laboratory used to prepare foods and beverages within a 1 g tolerance). The remaining energy was consumed via foods and beverages prepared by the participants at home with guidance from a registered dietitian (Supplementary Table S1).

During the 16-week weight-loss intervention period, each participant was prescribed a WSEP containing 750 kcal/day less than their estimated total energy requirement (Table 1) [17]. The energy deficit was achieved by removing non-protein foods and beverages (e.g., juices, butter, sodas, salad dressings) from each participant’s 3-week prescribed weight-maintenance baseline period menu (65% from carbohydrate and 35% from fat). Participants were randomized to consume additional select foods and beverages containing either 0.7 g carbohydrate/kg/day (CON; 0.8 g protein/kg/day in total) or 0.7 g protein/kg/day from milk protein isolate (MPI; 1.5 g protein/kg/day in total; Idaho Milk Products, Inc. Jerome, ID) (Supplementary Table S2). Participants in the CON and MPI group consumed the same prescribed WSEP and pre-prepared foods and beverages; however, a combination of carbohydrate sources (primarily maltodextrin) and milk protein isolate were isocalorically exchanged. A registered dietitian, using ProNutra software (Viocare Inc., Princeton, NJ), developed the individualized 7-day rotating menus for the 3-week prescribed weight-maintenance baseline period (Supplementary Table S1) and the intervention (Supplementary Table S3), and the foods and beverages containing carbohydrate or milk protein isolate (Supplementary Table S4). The study dietitian and other research staff members also contacted participants weekly in person, by e-mail, and/or by phone to encourage compliance with the prescribed menus.

### 2.4. Cardiometabolic Health Risk Factors

Fasting blood samples were collected, processed, and aliquots pipetted into cryovials for storage at −80 °C, as described [18] at the pre-study week, baseline week 3, and intervention week 16. Samples were thawed to assess plasma glucose, insulin, total cholesterol, high-density lipoprotein cholesterol, and triglyceride concentrations. Low-density lipoprotein cholesterol concentrations were calculated using the Friedewald formula [19]. Plasma glucose, lipids, and lipoproteins concentrations were determined by an oxidase method on a COBAS Integra 400 analyzer (Roche Diagnostic Systems, Indianapolis, IN). Plasma insulin concentrations were determined using an electrochemiluminescence immunoassay method on the Elecsys 2010 analyzer (Roche Diagnostic Systems). The homeostatic model assessment (HOMA) was used to calculate insulin resistance (IR) and beta-cell function (β) while fasting [20]. Fasting morning reclining systolic and diastolic blood pressures were measured according to the guidelines outlined by the International Organization for Standardization [21].

### 2.5. Ambulatory Blood Pressure

Ambulatory 24 h blood pressures were measured using an automatic monitoring system (Ambulo 2400, Tiba Medical, Portland, OR) on three separate days at baseline week 3 and intervention week 16. Automated blood pressure readings were made at 30-minute intervals during the day (08:00 h–17:00 h) and at 90-minute intervals during the night (17:00 h–08:00 h) [22]. Fluid intake was encouraged, and vigorous physical activity was prohibited for the three days prior to and the days of blood pressure assessments.

### 2.6. Body Composition

Fasting-state body mass was measured weekly to the nearest ±0.01 kg using a digital platform scale (model ES200L, Ohaus Corporation, Pine Brook, NJ). Standing height without shoes was measured to the nearest ±0.1 cm with a wall-mounted stadiometer during the pre-study week. Whole body lean (non-bone soft tissue) and fat tissue masses were measured by dual-energy X-ray absorptiometry (DXA, GE Lunar Prodigy with version 11.1 enCORE iDXA software, Madison, WI) during baseline week 3 and intervention week 16. Right mid-thigh and mid-calf cross-sectional areas, muscle areas, subcutaneous fat areas, and intramuscular adipose tissue (IMAT) areas were measured using magnetic resonance imaging (MRI, 3T General Electric Signa HDx system), as described [23]. Abdominal MRIs at the L3-L4 vertebral disc were also taken due to high correlations with whole abdominal visceral adipose tissue (VAT) volume [24].

### 2.7. Statistical Analysis

Power calculations were completed a priori for two independent means to detect a differential response between groups from baseline week 3 to intervention week 16 (α = 0.05, 80% power, G*Power version 3.0.10, Kiel, Germany). Published research suggests that *n* = 15/group would statistically support a differential change in thigh muscle area ≥ 1.75 ± 1.63 cm^2^ with effect size d = 1.07 (2-tailed) [25]. Additional a priori power calculations were performed for blood pressure and triglyceride concentrations. Samples sizes of *n* = 22/group and *n* = 21/group would statistically support a greater reduction in blood pressure of 4 mm Hg (effect size d = 0.77, one-tail) [26] and a 20% greater reduction in triglycerides (effect size d = 0.8, one-tail) [27] respectively, over 16 weeks. Therefore, as the longest duration among the studies used in the power calculations, 16 weeks was chosen as the intervention duration.

A two-tailed independent *t*-test was used to test for differences between participant characteristics in the CON and MPI groups at the pre-study week and baseline week 3. The main effects of time and group-by-time interaction were assessed with 2 × 2 factor repeated-measures analysis of covariance (ANCOVA) (MIXED procedure; group: CON, MPI; time: baseline week 3 and intervention week 16) for body composition outcomes. A 2 × 5 factor repeated-measures analysis of variance (ANOVA) (MIXED procedure; group: CON, MPI; time: baseline week 3 and intervention weeks 4, 8, 12, and 16) was used for fasting cardiometabolic health risk factors. Results are presented as least-square means (LSmeans) ± standard error (SE) unless otherwise noted, to reflect that age and sex were used as covariates. The pre-study week assessments of cardiometabolic health were an additional covariate for their respective outcomes. Statistical analyses were performed using SAS (version 9.3; SAS Institute Inc, Cary, NC). Significances were determined with a Tukey–Kramer corrected *p* < 0.05.

The clinical laboratory manager, who was not involved in data collection or analysis, generated the random allocation sequence using the first generation on randomization.com and assigned participants to the intervention in a 1:1 allocation ratio. The investigators and participants could not foresee which group they were allocated to and were also blinded during the intervention. At the end of the study, the blinding was broken prior to data analysis because the outcomes were not likely to be influenced by lack of blinding. Attrition rates were comparable between groups. All pre-specified outcomes are reported.

## 3. Results

### 3.1. Participant Characteristics

**Figure 1:**
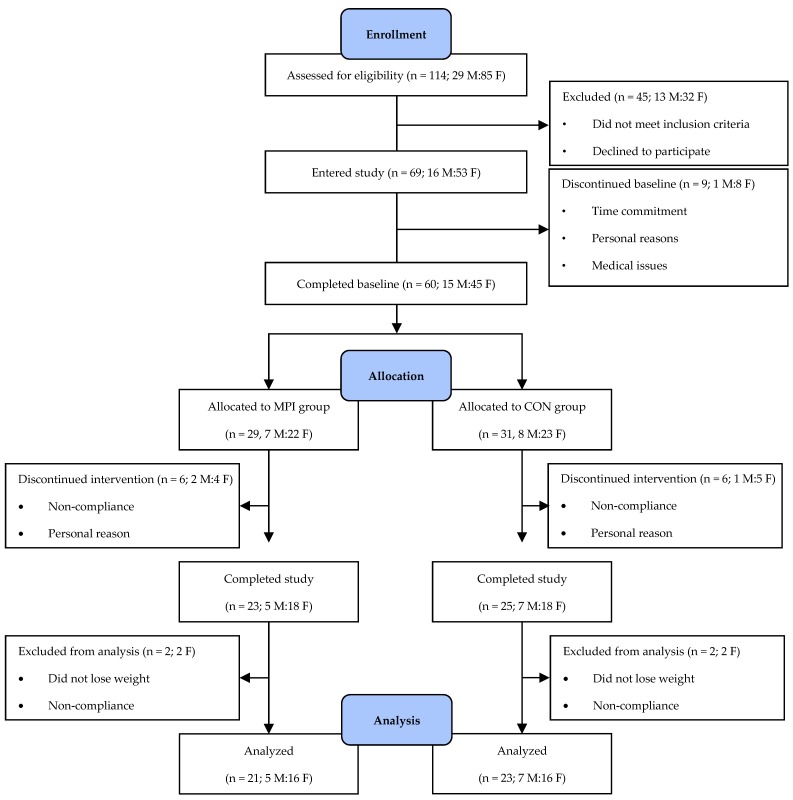
Consolidated Standards of Reporting Trials (CONSORT) flow diagram. CON, control intervention group consuming a Western-style eating pattern including an additional 0.7 g carbohydrate/kg/day. F, female, M, male. MPI, intervention group consuming a Western-style eating pattern including 0.7 g protein/kg/day from milk protein isolate.

Sixty-nine adults provided informed written consent to participate in this research study. Forty-eight participants completed the study and 44 were included in the final data analyses (Figure 1). Participant recruitment was stopped after reaching the targeted number of participants to account for drop-outs. During the pre-study week, habitual energy and macronutrient intakes were not different between the CON and MPI groups (Table 1). At baseline week 3, no differences were observed in age, BMI, cardiometabolic health risk factors, and whole body and tissue-specific morphological outcomes between the two groups, except for umbilicus circumference (Table 2 and Table 3).

### 3.2. Dietary Intake and Compliance

Participants in both groups were apparently compliant to their assigned diets. Each group reduced their body mass each week (Supplementary Figure S1), consistent with consuming an energy-restricted diet, and there were no differences in energy intake between groups, documented using menu checklists (Table 1). Menu checklists and 24 h urine urea nitrogen:creatinine ratio indicated that the total protein intakes were not different between groups pre-study and during baseline, but as designed, were lower for the CON group than the MPI group during the 16-week intervention (Supplementary Figure S2).

### 3.3. Cardiometabolic Health Risk Factors

Consuming a WSEP with select foods and beverages containing milk protein isolate differentially influenced changes in reclining systolic and diastolic blood pressures (group-by-time interactions, *p* < 0.05; Table 2). Reclining systolic blood pressure did not change from baseline for either group. Reclining diastolic blood pressure decreased from baseline in MPI, whereas CON did not change over time (Table 2). Independent of group, reclining diastolic blood pressure (−3 ± 1 mm Hg) decreased over time (main effects of time, *p* < 0.05; Table 2), whereas systolic blood pressure did not.

Triglyceride concentrations decreased more in the MPI groups compared the CON group (−29 ± 6 versus −42 ± 7 mg/dL) (Table 2). Among all participants over time, morning fasting plasma insulin (−4.6 ± 1.1 μU/mL), glucose (−3 ± 1 mg/dL), total cholesterol (−21 ± 3 mg/dL), and low-density lipoprotein cholesterol (−17 ± 2 mg/dL) concentrations, and total cholesterol:high-density lipoprotein cholesterol ratio (−0.79 ± 0.12) each decreased (main effects of time, *p <* 0.05). High-density lipoprotein cholesterol concentration increased (2 ± 1 mg/dL) whereas HOMA-IR (−1.1 ± 0.2) and HOMA-β (−40 ± 8) each decreased over time, independent of group (main effects of time, *p* < 0.05) (Table 2).

### 3.4. Ambulatory Blood Pressure

**Figure 2:**
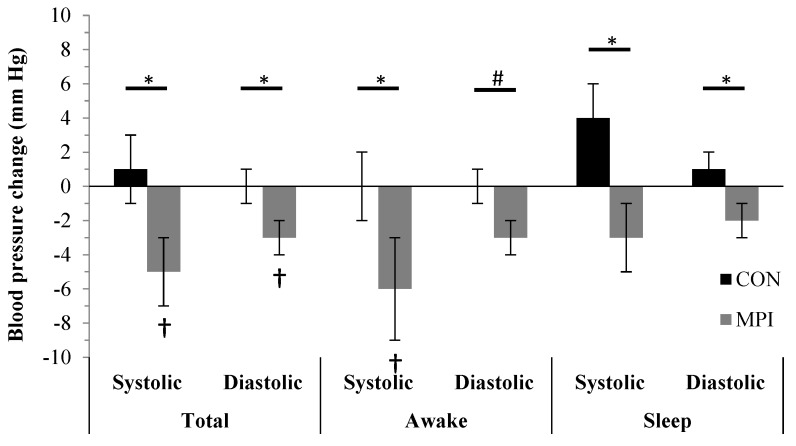
Changes in ambulatory blood pressures from baseline week 3 to intervention week 16 in the CON and MPI groups. Awake, waking hours blood pressure collected from 08:00 to 17:00 h in 30-minute intervals. CON, control intervention group consuming a Western-style eating pattern including and additional 0.7 g carbohydrate/kg/day. MPI, intervention group consuming a Western-style eating pattern including 0.7 g protein/kg/day from milk protein isolate. Sleep, sleeping hours blood pressure collected from 17:00 to 08:00 h in 90-minute intervals. Total, 24 h ambulatory blood pressure. * indicates a group-by-time interaction, *p* < 0.05. † indicates a change from baseline, *p* < 0.05. # indicates a time effect, *p* < 0.05.

Consuming a WSEP with milk protein isolate differentially influenced changes in total and sleeping hours systolic and diastolic blood pressures and waking hours systolic blood pressure (group-by-time interactions, *p* < 0.05; Table 2; Figure 2). In MPI, total systolic and diastolic blood pressures, and waking hours systolic blood pressure, decreased from baseline, whereas CON did not change (Table 2). Over time, waking hours diastolic blood pressure decreased (−2 ± 1 mm Hg), independent of group (main effects of time, *p* < 0.05; Table 2).

### 3.5. Body Composition

Consuming a WSEP with either carbohydrate or milk protein isolate did not influence changes in any of the whole-body tissue masses or changes in tissue specific outcomes (Table 3). Among all participants over time, whole body mass (−8.2 ± 0.4 kg), BMI (−3.0 ± 0.2 kg/m^2^), whole body lean mass (−1.2 ± 0.2 kg), whole body fat mass (−7.0 ± 0.4 kg), fat mass percentage (−4.6% ± 0.3%), lean mass index (−0.4 ± 0.1 kg/m^2^), fat mass index (−2.5 ± 0.2 kg/m^2^), natural waist circumference (−10.0 ± 0.9 cm), umbilicus circumference (−8.9 ± 0.7 cm), hip circumference (−7.3 ± 0.5 cm), and waist:hip ratio (−0.3 ± 0.01) each decreased and lean mass percentage (4.2% ± 0.3%) increased (main effects of time, *p* < 0.0001).

Total thigh area (−30 ± 2 cm^2^), thigh muscle area (−7 ± 1 cm^2^), thigh subcutaneous fat area (−22 ± 2 cm^2^), thigh IMAT area (−1 ± 0 cm^2^), and thigh IMAT:muscle area ratio (−0.5% ± 0.2%) each decreased (main effects of time, *p* < 0.01). Total calf area (−7 ± 1 cm^2^), calf muscle area (−3 ± 0 cm^2^), calf subcutaneous fat area (−3 ± 0 cm^2^), calf IMAT area (−1 ± 0 cm^2^), and calf IMAT:muscle area ratio (−0.8% ± 0.1%) each decreased (main effects of time, *p* < 0.0001). Thigh IMAT:subcutaneous fat area ratio (1.6% ± 0.4%) and calf IMAT:subcutaneous fat area ratio (−0.5% ± 0.3%) did not change over time. Abdominal VAT (−37 ± 6 cm^2^), VAT percent (−2.5% ± 0.6%), subcutaneous fat (−64 ± 7 cm^2^), and subcutaneous fat percent (−2.7% ± 0.6%) each decreased over time.

## 4. Discussion

This study was designed to assess the effects of exchanging carbohydrate (primarily maltodextrin) with milk protein isolate incorporated into foods and beverages typically consumed with a WSEP on weight-loss-induced changes in cardiometabolic health risk factors and whole body mass, fat mass, and lean mass in middle-aged adults classified as overweight or obese. In accordance with our hypothesis, compared to consuming a WSEP with foods and beverages containing primarily maltodextrin, consuming foods and beverages containing milk protein isolate improved fasting reclining and ambulatory blood pressures after weight loss. However, contrary to our hypothesis, consuming milk protein isolate did not result in less lean mass loss or greater fat mass reductions. The results from this study indicate that substituting carbohydrate with milk protein isolate may be an effective dietary strategy for improving blood pressure during weight loss.

We uniquely prescribed a WSEP, an “unhealthy” diet, to participants before (3-week baseline) and during (16-week intervention) weight loss. During the weight loss period, participants consumed select foods and beverages containing either milk protein isolate or an isocalorically matched quantity of carbohydrate (primarily maltodextrin). This study distinguishes itself from previous research in that the carbohydrate and milk protein isolate were not consumed as shakes or bars to supplement the diet. Rather, they were incorporated as ingredients into select foods and beverages in the diet by a registered dietitian in our metabolic kitchen. While participants were responsible for purchasing the majority of items on their prescribed menus, the foods and beverages containing the carbohydrate and milk protein isolate ingredients were distributed weekly to the participants. While this dietary control approach is less rigorous than providing precisely portioned amounts of all menu items, expected reductions in body mass occurred over time for both groups, consistent with consuming an energy deficit of 750 kcal [28,29]. The primary objective was to include a potentially health promoting ingredient (i.e., milk protein isolate) into an (“unhealthy”) eating pattern typically consumed by most Americans, but with weight loss. Consequently, the MPI group consumed a higher protein diet (1.5 g protein/kg/day) than the CON group (0.8 g protein/kg/day). Consistent with this design, we reported lower urine urea nitrogen/creatinine ratio in CON versus MPI during the intervention. Another novelty of this study was measurement of thigh and calf muscle quality (muscle size, IMAT, and IMAT/muscle ratio), and abdominal adiposity using MRI in addition to DXA. We observed comparable changes among whole body and tissue-specific outcomes, supporting consistency between the two measurement methods.

In our study, the MPI group improved their blood pressures after 16 weeks of weight loss while the CON group did not. Results from the National Health and Nutrition Examination Survey [30], the Rotterdam Study [31], and the Maine-Syracuse Longitudinal Study [32], showed that higher dairy consumption was predictive of lower blood pressure. The results from two meta-analyses of randomized controlled trials with European [33] and Asian [34] cohorts indicated that milk tripeptides reduced blood pressure, which may inhibit angiotensin-converting enzyme, vasodilation, and reduce sympathetic nervous activity [35]. Conversely, maltodextrin is a glucose polymer powder that may worsen hypertension-related gene expression [36]. In one randomized controlled trial, adults with upper-range prehypertension and grade 1 hypertension consuming a higher protein diet (~25% of total energy intake) versus a lower protein diet (~15%, at the expense of maltodextrin) had lower office and daytime blood pressures [37]. As a result of the milk protein isolate, the MPI group also consumed more calcium—and possibly other bioactive compounds, such as potassium—known blood pressure reducers [36]. However, distinguishing the specific direct or indirect causes for how milk protein isolate effects blood pressure cannot be determined from this study because we utilized an ingredient containing a myriad of nutrients—calcium, potassium, vitamin D, proteins, etc. We report here that substituting carbohydrate with milk protein isolate, resulting in a lower (0.8 g/kg/day) and higher (1.5 g/kg/day) protein diet, may be an effective dietary strategy for improving blood pressure after weight loss.

The greater improvement in serum triglyceride concentrations observed in the MPI compared to the CON group is consistent with multiple studies [38,39,40] using a higher versus lower protein intake design. Generally, higher protein intakes are achieved by substituting the additional protein for either all or most carbohydrates. In the case of this study, milk protein isolate was substituted for all carbohydrate, primarily maltodextrin. The simultaneous increase in protein and decrease in carbohydrate is generally recognized to result in lowering triglyceride concentrations [41]. However, it is unclear whether the improvements observed in this study can be attributed specifically to milk protein or to the higher protein diet.

We did not observe a differential effect on total body mass loss or on the reductions in fat and lean masses. These results are contrary to a meta-analysis of 37 randomized controlled trials assessing the effect of dairy consumption on body weight and body composition with and without energy restriction [42]. After energy restriction, higher dairy intake promoted greater body weight and fat mass losses but had no effect on lean body mass [42]. One possible explanation for the null results of the current study may be that the potential effect of consuming a higher dairy diet, known as the “dairy effect”, may require over a year to manifest [12]. However, this seems unlikely as all of the trials with energy restriction in the meta-analysis had durations of 12 months or less [42]. Notably, each of the trials vetted and included with energy restriction used whole-dairy foods such as yogurt and fluid milk, rendering the results not directly comparable to our study. Only one trial out of the 37 utilized an isolate dairy protein source and this study was not a randomized controlled trial but a secondary analysis [43]. That study was also designed without energy restriction and the dairy source, milk protein concentrate, supplemented the participants’ diets. The lack of directly comparable published research underscores the novelty of our study to use milk protein isolate as an ingredient in foods and beverages.

By exchanging carbohydrate with a dairy protein source, milk protein isolate, we provided the MPI group with an additional 0.7 g protein/kg/day (1.5 g/kg/day in total). Our results are contrary to several previous meta-analyses that reported consuming a higher protein diet reduced lean body mass lost after diet-induced weight loss [29,44,45,46]. However, caution is warranted to not directly compare the results from our study with the meta-analyses, in part because none of the trials vetted and included in the meta-analyses used milk protein isolate as a major protein source. In light of this, we reviewed another meta-analysis [47] on the effects of protein supplementation on body composition changes, albeit all performed some form of exercise. The authors [47] reported that consuming a protein supplement while engaged in an exercise training program resulted in participants consuming a higher protein diet and having a greater increase in lean body mass compare to the control group. Exercise may be the primary stimulus that influences changes in lean mass. Consuming a protein supplement to achieve a higher protein diet may potentiate the response [48]. The addition of an exercise stimulus in the current study may have yielded results more comparable to the meta-analysis. However, in this case, only 3 of the 22 randomized controlled trials vetted assessed the effect of milk supplementation and none used milk protein isolate. To our knowledge, this is the only study to directly test the effect of consuming milk protein isolate as an ingredient in foods and beverages that conform to a WSEP, rendering direct comparisons to existing literature unadvisable.

The “quarter lean mass rule” is often invoked to indicate the expected proportion of body weight lost as lean body mass after dietary energy restriction [49]. When dietary energy restriction is combined with exercise, the proportion of body weight lost as lean mass decreases [49,50]. A similar phenomena is documented when an energy-restricted high-protein diet is consumed relative to a lower protein diet [29,44]. We anticipated that the CON group would lose ~25% of their body mass as lean mass while the MPI group would lose less than 25%. However, both groups lost ~15% of body mass as lean mass. A 15% loss as lean mass is within the documented range for participants of similar body fatness and degree of energy restriction who are not exercising or consuming a “high” protein diet [49].

The non-differential effects of fat mass loss may be a result of the calcium content in the diets. Calcium from dairy sources may act as an anti-obesity bioactive by increasing fecal fat excretion [51], fat oxidation [52], thermogenesis [53,54], and decreasing fat absorption [55]. These effects, however, may only manifest up to a calcium intake threshold of 500–700 mg/day [35]. The 600 mg/day of calcium provided to the CON group was right at the threshold; therefore, the ~1500 mg/day more calcium provided to the MPI group than the CON group would not enhance the potential effect on fat mass loss. In a similar study, fat mass loss between high and normal protein intakes (1600 and 600 mg/day Ca, respectively) were also not different. These results and ours are consistent with the concept of a calcium threshold [56]. However, both the current study and the study above [56] used a whole foods approach, thus a limitation of the study is the inability to separate the effects of protein and other bioactive compounds, including calcium.

## 5. Conclusions

This study assessed the effects of replacing carbohydrate with milk protein isolate in an energy-restricted WSEP on cardiometabolic health risk factors and body composition. Altering habitual, typically unhealthy, eating patterns to more closely resemble a healthy dietary pattern has proven difficult to achieve amongst the general US population [3]. Alternative dietary approaches that reduce the barrier to consuming health-promoting ingredients and eating patterns are needed. For apparently healthy adults who are overweight or moderately obese without chronic diseases, engaging in a weight loss program will improve both select cadiometabolic health and metabolic syndrome risk factors and reduce their risk for developing obesity-related comorbidities [16]. For those who may find it difficult to deviate from a WSEP, replacing a portion of their carbohydrate with foods and beverages containing milk protein isolate may be an effective dietary strategy to reduce blood pressure and improve triglyceride concentrations after weight loss.

## Figures and Tables

**Table 1:** Participant’s habitual, prescribed, and actual nutritional intakes in the control (CON) and milk protein isolate (MPI) groups ^1^.

	CON			MPI		
Parameters	Pre-Study Week	Prescribed	Actual	Pre-Study Week	Prescribed	Actual
Energy (kcal/day)	2142 ± 119	1684 ± 70	1620 ± 64	2112 ± 148	1732 ± 75	1682 ± 75
Fat (g/day)	87 ± 6	55 ± 2	—	85 ± 7	56 ± 2	—
Carbohydrate (g/day)	260 ± 14	227 ± 10 ^a^	—	256 ± 16	173 ± 9 ^b^	—
Protein (g/day)	83 ± 5	68 ± 2 ^a^	65 ± 2 ^a^	83 ± 6	134 ± 4 ^b^	130 ± 4 ^b^
Protein ^2^ (g/kg/day)	0.98 ± 0.6	0.80 ± 0.0 ^a^	0.76 ± 0.01 ^a^	0.92 ± 0.05	1.50 ± 0.0 ^b^	1.44 ± 0.01 ^b^

^1^ values are presented as means ± standard error (SE); *n* = 21 for the CON group and *n* = 23 for the MPI group. An unpaired t-test (TTEST procedure, SAS version 9.3; SAS Institute) was used to test for differences between groups at the same time point. Different letter superscripts indicate a difference between groups within a corresponding column, *p* < 0.05. Actual = actual intake of participants consuming the prescribed Western-style eating pattern as assessed using the daily menu checklists with the Nutrition Data System for Research software. CON = control intervention group consuming a Western-style eating pattern including 0.7 g carbohydrate/kg/d from maltodextrin. MPI = intervention group consuming a Western-style eating pattern including 0.7 g protein/kg/d from milk protein isolate. Prescribed = the prescribed Western-style eating pattern developed in ProNutra software as assessed using Nutrition Data System for Research software. Pre-study week = participants habitual, self-chosen, diets as assessed using data from 3 days of food records with Nutrition Data System for Research software. ^2^ Relative protein intake was calculated using baseline body weight.

**Table 2:** Changes in cardiometabolic health risk factors in the CON and MPI groups after consuming an energy-restricted diet for 16 weeks ^1^.

		Time (weeks)		
Parameters	Group	Baseline	16	Change
**Fasting cardiometabolic health risk factors**				
Insulin ^2^ (pmol/L)	CON	74.3 ± 7.6	46.5 ± 7.6	−27.8 ± 6.9
	MPI	86.1 ± 7.6	50.0 ± 7.6	−36.1 ± 6.9
Glucose ^2^ (mmol/L)	CON	5.05 ± 0.11	4.88 ± 0.11	−0.17 ± 0.06
	MPI	5.22 ± 0.11	5.00 ± 0.11	−0.22 ± 0.06
Total cholesterol ^2^ (mmol/L)	CON	4.47 ± 0.16	4.00 ± 0.16	−0.47 ± 0.10
	MPI	4.47 ± 0.16	3.85± 0.16	−0.75 ± 0.10
HDL-C ^2^ (mmol/L)	CON	1.16 ± 0.05	1.22 ± 0.05	0.05 ± 0.03
	MPI	1.09 ± 0.08	1.14 ± 0.08	0.05± 0.03
LDL-C ^2^ (mmol/L)	CON	2.72 ± 0.13	2.33 ± 0.13	−0.39 ± 0.08
	MPI	2.72 ± 0.16	2.73 ± 0.16	−0.47 ± 0.08
Total cholesterol:HDL-C ^2^	CON	4.10 ± 0.22	3.46 ± 0.22	−0.63 ± 0.15
	MPI	4.37 ± 0.24	3.45 ± 0.24	−0.92 ± 0.15
Triglycerides ^2,3^ (mmol/L)	CON	1.32 ± 0.10	0.99 ± 0.10	−0.33 ± 0.07
	MPI	1.41 ± 0.10	0.94 ± 0.10	−0.47 ± 0.08
HOMA-IR ^2^	CON	2.42 ± 0.27	1.47 ± 0.27	−0.95 ± 0.25
	MPI	2.97 ± 0.29	1.64 ± 0.29	−1.33 ± 0.26
HOMA-β ^2^	CON	140 ± 13	104 ± 13	-37 ± 11
	MPI	139 ± 14	95 ± 14	−44 ± 11
Reclining systolic ^3^ (mm Hg)	CON	116 ± 2	118 ± 2	2 ± 2
	MPI	116 ± 2	111 ± 2	−4 ± 2
Reclining diastolic ^2,3^ (mm Hg)	CON	75 ± 2	75 ± 2	−0 ± 1
	MPI	78 ± 2	73 ± 2	−5 ± 1
**Ambulatory blood pressures (mm Hg)**				
Total systolic ^3^	CON	129 ± 2	131 ± 2	1 ± 2
	MPI	130 ± 2	125 ± 2	−5 ± 2
Total diastolic ^3^	CON	76 ± 2	77 ± 2	0 ± 1
	MPI	78 ± 2	74 ± 2	−3 ± 1
Awake systolic ^3^	CON	133 ± 2	133 ± 2	0 ± 2
	MPI	133 ± 2	128 ± 2	−6 ± 3
Awake diastolic ^2^	CON	79 ± 2	79 ± 2	0 ± 1
	MPI	80 ± 2	77 ± 2	−3 ± 1
Sleep systolic ^3^	CON	116 ± 2	121 ± 2	4 ± 2
	MPI	118 ± 3	115 ± 3	−3 ± 2
Sleep diastolic ^3^	CON	66 ± 2	68 ± 2	1 ± 1
	MPI	68 ± 2	66 ± 2	−2 ± 1

^1^ Values are presented as least-squares mean ± standard error, *n* = 23 for the CON group and *n* = 21 for the MPI group. An unpaired *t*-test (TTEST procedure, SAS version 9.3; SAS Institute) was used to test for differences baseline characteristics. A repeated-measure analysis of variance (ANOVA) (MIXED procedure, SAS version 9.3; SAS Institute) was used to test for main effects of time and group-by-time interactions. β, beta cell function; CON, control intervention group consuming a Western-style eating pattern including 0.7 g carbohydrate/kg/d; HDL-C, high-density lipoprotein cholesterol; HOMA, Homeostatic model assessment; IR, insulin resistance; LDL-C, low-density lipoprotein cholesterol; MPI, intervention group consuming a Western-style eating pattern including 0.7 g protein/kg/day from milk protein isolate. ^2^ Main effect of time, *p* < 0.05. ^3^ Group-by-time interaction, *p* < 0.05.

**Table 3:** Changes in anthropometrics and body composition in the CON and MPI groups after consuming an energy-restricted diet for 16 weeks ^1^.

		Time (Weeks)		
Parameters	Group	Baseline	16	Change
Age (years)	CON	52 ± 1	—	—
	MPI	53 ± 2	—	—
Body mass ^2^ (kg)	CON	87.1 ± 2.5	79.2 ± 2.5	−7.9 ± 0.6
	MPI	91.6 ± 2.8	83.1 ± 2.8	−8.5 ± 0.6
BMI ^2^ (kg/m^2^)	CON	30.3 ± 0.7	27.4 ± 0.7	−2.9 ± 0.2
	MPI	31.0 ± 0.7	28.0 ± 0.7	−3.1 ± 0.3
Natural waist circumference ^2^ (cm)	CON	103.3 ± 1.8	93.5 ± 1.8	−9.8 ± 1.2
	MPI	106.0 ± 1.9	95.8 ± 1.9	−10.2 ± 1.3
Umbilicus circumference ^2^ (cm)	CON	102.9± 1.8	95.0 ± 1.8	−7.9 ± 0.9
	MPI	109.0 ± 2.0	99.2 ± 2.0	−9.8 ± 1.0
Hip circumference ^2^ (cm)	CON	109.9 ± 1.7	103.0 ± 1.7	−6.9 ± 0.7
	MPI	113.8 ± 1.9	106.2 ± 1.9	−7.7 ± 0.7
Natural waist:Hip ratio^2^	CON	0.94 ± 0.01	0.91 ± 0.01	−0.03 ± 0.01
	MPI	0.94 ± 0.01	0.91 ± 0.01	−0.03 ± 0.01
**Whole body**				
Lean mass ^2^ (kg)	CON	49.6 ± 1.0	48.5 ± 1.0	−1.1 ± 0.2
	MPI	51.7 ± 1.2	50.5 ± 1.2	−1.3 ± 0.3
Lean mass ^2^ (%)	CON	56.8 ± 1.0	60.2 ± 1.0	4.3 ± 0.4
	MPI	56.6 ± 1.1	60.8 ± 1.1	4.1 ± 0.5
Fat mass ^2^ (kg)	CON	34.6 ± 1.7	28.8 ± 1.7	−6.8 ± 0.5
	MPI	36.9 ± 2.0	29.6 ± 2.0	−7.2 ± 0.6
Fat mass ^2^ (%)	CON	39.9 ± 1.1	35.2 ± 1.1	−4.7 ± 0.4
	MPI	40.1 ± 1.2	35.6 ± 1.2	−4.5 ± 0.5
Lean mass index ^2^ (kg/m^2^)	CON	17.1 ± 0.2	16.7 ± 0.2	−0.4 ± 0.1
	MPI	17.4 ± 0.3	16.9 ± 0.3	−0.5 ± 0.1
Fat mass index ^2^ (kg/m^2^)	CON	12.2 ± 0.5	9.7 ± 0.5	−2.5 ± 0.2
	MPI	12.6 ± 0.6	10.0 ± 0.6	−2.6 ± 0.2
**Right mid-thigh areas**				
Total cross section ^2^ (cm^2^)	CON	237 ± 9	209 ± 9	−28 ± 3
	MPI	259 ± 9	228 ± 9	−32 ± 3
Muscle ^2^ (cm^2^)	CON	122 ± 3	115 ± 3	−7 ± 1
	MPI	127 ± 3	120 ± 3	−7 ± 1
Subcutaneous fat ^2^ (cm^2^)	CON	97 ± 8	77 ± 8	−20 ± 2
	MPI	113 ± 9	90 ± 9	−23 ± 2
IMAT ^2^ (cm^2^)	CON	11 ± 1	10 ± 1	−1 ± 0
	MPI	12 ± 1	11 ± 1	−1 ± 0
IMAT:Muscle ratio ^2^ (%)	CON	9.2 ± 0.8	8.5 ± 0.8	−0.6 ± 0.2
	MPI	9.5 ± 0.8	9.1 ± 0.8	−0.3 ± 0.3
IMAT:Subcutaneous fat ratio ^2^ (%)	CON	12.8 ± 1.3	14.1 ± 1.3	1.3 ± 0.5
	MPI	13.0 ± 1.4	14.9 ± 1.4	1.9 ± 0.6
**Right mid-calf areas**				
Total cross section ^2^ (cm^2^)	CON	116 ± 4	110 ± 4	−6 ± 1
	MPI	126 ± 4	118 ± 4	−8 ± 1
Muscle ^2^ (cm^2^)	CON	71 ± 2	68 ± 2	−3 ± 1
	MPI	73 ± 2	70 ± 2	−3 ± 1
Subcutaneous fat ^2^ (cm^2^)	CON	30 ± 3	27 ± 3	−3 ± 1
	MPI	37 ± 3	33 ± 3	−4 ± 1
IMAT ^2^ (cm^2^)	CON	7 ± 1	6 ± 1	−1 ± 0
	MPI	8 ± 1	7 ± 1	−1 ± 0
IMAT:Muscle ratio ^2^ (%)	CON	9.9 ± 0.8	9.2 ± 0.8	−0.6 ± 0.1
	MPI	10.2 ± 0.9	9.2 ± 0.9	−1.0 ± 0.2
IMAT:Subcutaneous fat ratio (%)	CON	26.2 ± 2.6	25.6 ± 2.6	−0.7 ± 0.4
	MPI	25.3 ± 2.9	25.0 ± 2.9	−0.3 ± 0.4
**Abdominal L3–L4 vertebrae**				
Subcutaneous fat ^2^ (cm^2^)	CON	292 ± 25.06	229 ± 25	−63 ± 9
	MPI	333 ± 26.87	268 ±27	−65 ± 9
VAT ^2^ (cm^2^)	CON	136 ± 11.57	100 ± 12	−36 ± 8
	MPI	145 ± 12.34	107 ± 12	−38 ± 8
Subcutaneous fat ^2^ (%)	CON	40.0 ± 2.2	37.0 ± 2.2	−3.0 ± 0.9
	MPI	41.1 ± 2.3	38.7 ± 2.3	−2.4 ± 0.9
VAT ^2^ (%)	CON	18.9 ± 1.4	16.3 ± 1.4	−2.7 ± 0.8
	MPI	17.9 ± 1.5	15.7 ± 1.5	−2.3 ± 0.8

^1^ Age is presented as mean ± standard err. All other values are presented as LSmean ± SE, *n* = 21 for the CON group and *n* = 23 for the MPI group. An unpaired *t*-test (TTEST procedure, SAS version 9.3; SAS Institute) was used to test for differences baseline characteristics. A repeated-measure ANOVA (MIXED procedure, SAS version 9.3; SAS Institute) was used to test for main effect of time and group-by-time interaction. CON, control intervention group consuming a Western-style eating pattern including 0.7 g carbohydrate/kg/day; G × T, group-by-time interaction; IMAT, intramuscular adipose tissue; MPI, intervention group consuming a Western-style eating pattern including 0.7 g protein/kg/day from milk protein isolate; VAT, visceral adipose tissue. ^2^ Main effect of time, *p* < 0.05.

## Supplementary Materials

The following are available online at https://www.mdpi.com/2072-6643/12/3/851/s1, Figure S1: Weekly bodyweight in the CON and MPI groups, Figure S2: Urinary urea nitrogen to creatinine ratios in the CON and MPI groups, Table S1: Example menu for a~2350 kcal Western-style eating pattern consumed during the 3-week baseline period, Table S2: Nutritional composition of the milk protein isolate; Table S3: Example menu for a 1600 kcal Western-style eating pattern consumed during the 16-week intervention period, Table S4: The ingredient list and gram weights for the select foods and beverages that contained milk protein isolate or maltodextrin during the intervention for a 1600 kcal menu.
