# Supplementary material for: Incorporating Milk Protein Isolate into an Energy-Restricted Western-Style Eating Pattern Augments Improvements in Blood Pressure and Triglycerides, but Not Body Composition Changes in Adults Classified as Overweight or Obese: A Randomized Controlled Trial
Source: Nutrients. 2020 Mar 22;12(3):851. doi: 10.3390/nu12030851 (PMC7146470; doi:10.3390/nu12030851)
Supplement: Supplementary file 1 [file nutrients-12-00851-s001.pdf]

Supplemental Table 1. Example menu for a ~2350 kcal Western-style eating pattern consumed during the 3-week baseline period

	Quantity	Unit
**Day 1**		
**Breakfast**		
Kroger Southern Style Biscuit	2	each
Butter*	5	gram
Jimmy Dean Fully Cooked Sausage Patties	43	gram
Pineapple, canned in heavy syrup	1/2	cup
Welch's Essentials Juice Cocktail	10	fl oz
**Lunch**		
Michelina's Authentic Cheese Manicotti with Marinara Sauce	1	meal (213 gram)
Garden Salad		
Iceburg Lettuce, shredded	3/4	cup
Cucumber, sliced with peel	2	slices (1/4 inch each)
Red Cherry Tomato	2	each
Baby Carrots	2	each
Shredded Cheddar Cheese, 2% Milkfat	8	gram
Ranch Dressing	1	Tbsp
Croutons	7	gram
Strawberries, halves	1 1/2	cup
Minute Maid Lemonade	10	fl oz
**Mid-Afternoon Snack**		
Zone Perfect Bar*	1	serving (37 gram)
**Dinner**		
Pork Loin, raw weight	95	gram
Sweet Potato, without peel	1	medium
Mini Marshmallows*	10	each
Frozen Broccoli	1	cup
Kraft American Cheese Singles	9.5	gram
King's Hawaiian Honey Wheat Roll	1	each
Butter*	5	gram
Jello Gelatin Dessert (any flavor)	1	each
Regular Cool Whip	1/4	cup
Sprite	1	can (12 fl oz)
**Day 2**		
**Breakfast**		
Grands Mini Buttermilk Biscuits*	2	each
Butter*	10	gram
Kroger Blended Yogurt (any flavor)	1	container (170 gram)
Grape Nuts Cereal	1/4	cup
Welch's Essentials Juice Cocktail	10	fl oz
**Lunch**		
Campbell's Chunky Chicken Broccoli Cheese & Potato Soup	320	gram
Healthy Life 100% Whole Wheat Bread	1	slice
Corn	1/2	cup
Butter*	15	gram
Apple Pear Fruit Crisp	1	each
Soda	1	can (12 fl oz)
**Mid-Afternoon Snack**		
Granola*		
Kroger 100% Whole Grain Oats	19	gram
Slivered almonds	6	gram
Vital wheat gluten	8	gram
Log Cabin NSA pancake syrup	4	gram
Splenda	1	gram

Dried banana	5	gram
**Dinner**		
Frozen Chicken Breast, fully cooked	90	gram
White Rice, cooked	1/2	cup
Carrots, frozen, sliced	1/2	cup
Butter*	15	gram
<u>Garden Salad</u>		
Iceburg Lettuce, shredded	3/4	cup
Cucumber, sliced with peel	2	slices, 1/4" each
Red Cherry Tomatoes	2	each
Baby Carrots	2	each
Shredded Cheddar Cheese, 2% Milkfat	20	gram
Ranch Dressing	1 1/2	Tbsp
Jello-O Sugar Free Pudding	1	each
Lemonade	16	fl oz
**Day 3**		
**Breakfast**		
Kroger Homestyle Frozen Waffles	2	each
Butter*	20	gram
Original Aunt Jemima Syrup	3	Tbsp
Land O Frost Canadian Bacon Natural Hickory Smoked	43	gram
Mixed Fruit, no added sugar	1	each (1/2 cup)
Welch's Essentials Juice Cocktail	8	fl oz
**Lunch**		
Stouffer's Macaroni & Cheese (frozen)	148	gram
Campbell's Kettle Soups, Tomato & Sweet Basil Bisque	180	gram
Apple Cinnamon Fruit Crisp	1	each
Soda	1	can (12 fl oz)
**Mid-Afternoon Snack**		
Skim Milk	12	fl oz
Oreo Cookies	2	each
**Dinner**		
Beef Tenderloin, broiled*	80	gram
Heinz Home Style Savory Beef Gravy	1/4	cup
Ore Ida Steam & Mash Cut Potatoes	3/4	cup
Cut Green Beans, No Salt Added	1/2	cup
King's Hawaiian Whole Wheat Roll	1	each
Vividly Vanilla Ice Cream, no added sugar	1/3	cup
Butter*	5	gram
Jellybeans*	15	each
**Day 4**		
**Breakfast**		
Grands Mini Buttermilk Biscuits*	2	each
Butter*	10	gram
Kroger Blended Yogurt (any flavor)	1	container (170 gram)
Grape Nuts	1	Tbsp
Apple Pear Fruit Crisp	1	each
Welch's Essentials Juice Cocktail	10	fl oz
**Lunch**		
Campbell's Chunky Chicken Corn Chowder	~1	cup (266 gram)
Healthy Life 100% Whole Wheat Bread	1	slice
Shredded Cheese, 2% Milkfat	16	gram
Pimento*	6	gram
Heinz Real Mayonnaise*	2	Tbsp
Carrots, sliced frozen	1/2	cup
Butter*	15	gram
Teddy Grahams*	5	each

Vanilla Frosting*	10	gram
Jellybeans*	8	each
Pepsi	1	can (12 fl oz)
**Mid-Afternoon Snack**		
Zone Perfect Bar*	1	serving (37 gram)
**Dinner**		
Barilla Plus Rotini Pasta, dry weight	20	gram
Prego Meatless Spaghetti Sauce	1/2	cup
Ground Beef, 90% lean, raw weight	85	gram
Parmesan Cheese	3	Tbsp
Coles Sliced Garlic Bread	1/2	slice
Lettuce, shredded	3/4	cup
Croutons*	7	gram
Ken's Steakhouse Light Creamy Caesar Dressing	1	Tbsp
Jello Gelatin Dessert	1	each
Cool Whip Topping	3 1/2	Tbsp
Lemonade	6	fl oz
**Day5**		
**Breakfast**		
Kroger Southern Style Biscuit	2	each
Butter*	10	gram
Jimmy Dean Fully Cooked Sausage Patties	1	patty (43 gram)
Welch's Essentials Juice Cocktail	8	fl oz
**Lunch**		
Michelina's Authentic Cheese Manicotti with Marinara Sauce	1	meal (213 gram)
Garden Salad		
Iceburg Lettuce, shredded	3/4	cup
Cucumber, sliced with peel	2	slices (1/4 inch each)
Red Cherry Tomato	2	each
Baby Carrots	2	each
Shredded Cheddar Cheese, 2% Milkfat	9	gram
Croutons*	7	gram
Ranch Dressing	1	Tbsp
Watermelon, cubed	1/2	cup
Jell-O Sugarfree Pudding	1	each
Jellybeans*	10	each
Soda	1	can (12 fl oz)
**Mid-Afternoon Snack**		
Granola*		
Kroger 100% Whole Grain Oats	19	gram
Slivered almonds	6	gram
Vital wheat gluten	8	gram
Log Cabin NSA pancake syrup	4	gram
Splenda	1	gram
Dried banana	5	gram
**Dinner**		
Kentucky Legend Hickory Smoked Hamsteak	112	gram
Kroger Roasted Redskin Potatoes, frozen	1/2	cup
King's Hawaiian Whole Wheat Roll	1	each
Butter*	10	gram
Peas, frozen	1	cup
Lemonade	8	fl oz
Jellybeans*	8	each
**Day 6**		
**Breakfast**		
Kroger Homestyle Frozen Waffles	10	gram
Butter*	10	gram

Original Aunt Jemima Syrup	2 1/2	Tbsp
Land O Frost Canadian Bacon Natural Hickory Smoked	43	gram
Mixed Fruit, no added sugar	1	each
Welch's Essentials Juice Cocktail	8	fl oz
**Lunch**		
Stouffers Macaroni & Cheese (frozen)	1/2	cup (118 gram)
Campbell's Kettle Soups, Tomato & Sweet Basil Bisque	~1 1/2	cup (330 gram)
Pineapple, canned in heavy syrup	1/2	cup
Pepsi	1	can (12 fl oz)
**Mid-Afternoon Snack**		
Skim Milk	10	fl oz
Oreo Cookies	3	each
**Dinner**		
Beef Burrito		
Ole Extreme Wellness Whole Wheat Tortilla	1	each
Tyson Grilled & Ready Seasoned Steak Strips	88	gram
Lettuce, shredded	1/2	cup
Shredded Cheddar cheese, 2% Milkfat	12	gram
Heinz Real Mayonnaise*	1	Tbsp
Southwestern Ranch Dressing*	16	gram
Tostitos Scoops	10	each
Salsa	2	Tbsp
Orange, fresh	1	each
Sprite	1	can (12 fl oz)
**Day 7**		
**Breakfast**		
Grands Mini Buttermilk Biscuits*	1	each
Butter*	5	gram
Kroger Blended Yogurt (any flavor)	1	container (170 gram)
Grape Nuts	1/4	cup
Apple Pear Fruit Crisp	1	container
Welch's Essentials Juice Cocktail	12	fl oz
**Lunch**		
Campbell's Chunky Chicken Corn Chowder	360	gram
Kraft American Cheese	19	gram
Cut Green Beans, No Added Salt	1	cup
Butter*	15	gram
Watermelon, raw	1/2	cup
Lemonade	12	fl oz
**Mid-Afternoon Snack**		
Soy Nuts*	28	gram
Diet Soda	1	can (12 fl oz)
**Dinner**		
Frozen Grilled Chicken Breast, fully cooked	80	gram
Potato, small baked	1	each
Oscar Mayer Turkey Bacon	1	slice
Sour Cream	1	Tbsp
Shredded Cheddar Cheese, 2% Milkfat	9	gram
Coles Sliced Garlic Bread	1/2	slice
Carrots, slice, frozen	1/2	cup
Butter*	15	gram
Vividly Vanilla Ice Cream, No Added Sugar	1/3	cup
Hot Fudge or Hot Caramel	1/2	Tbsp
Jellybeans*	8	each
Lemonade	8	fl oz

*Indicates foods were provided to participant

Supplemental Table 2. Example menu for a 1600 kcal Western-style eating pattern consumed during the 16-week intervention period

	Quantity	Unit
**Day 1**		
**Breakfast**		
Biscuit#	2	each
Gravy#	178	gram
Jimmy Dean Fully Cooked Sausage Links	21	gram
Skim milk	6	fl oz
**Lunch**		
Pepperoni Pizza		
Pizza Crust#	1	each
Rago Pizza Sauce	1/2	cup
Pepperoni	14	gram
Mozzarella Cheese	22	gram
Garden Salad		
Shredded lettuce, iceberg	3/4	cup
Cucumber, sliced with peel	2	slices, 1/4" each
Red Cherry Tomatoes	2	each
Baby Carrots	2	each
Ranch Dressing, fat free	1	Tbsp
Fruit Smoothie#	1	serving (250 gram)
**Mid-Afternoon Snack**		
Cliff Builder Bar*	1	serving (36 gram)
**Dinner**		
Pork Loin, raw weight	103	gram
Sweet Potato, boiled with no skin	1	medium
Mini-Marshmallows*	10	each
Frozen Broccoli	1	cup
Kraft American Cheese	10	gram
King's Hawaiian Honey Wheat Dinner Roll	1	each
Butter*	5	gram
**Day 2**		
**Breakfast**		
Blueberry Muffin*	2	each
Kroger Carbmaster Yogurt (any flavor)	1	each (170 gram)
Almonds, Blanched*	4	gram
Quaker Kretschmer Wheat Germ*	10	gram
Vanilla Drink#	~12	fl oz (297 gram)
Chocolate Flavoring Syrup*	10	gram
**Lunch**		
Corn Chowder#	1	serving
Healthy Life 100% Whole Wheat Bread	1	slice
Honey Ham	21	gram
American Cheese	19	gram
Diet Pepsi*	1	can (12 fl oz)
**Mid-Afternoon Snack**		
Granola*		
Kroger 100% Whole Grain Oats	19	gram
Slivered almonds	6	gram
Vital wheat gluten	8	gram
Log Cabin NSA pancake syrup	4	gram
Splenda	1	gram
Dried banana	5	gram
**Dinner**		
Frozen Chicken Breast, fully cooked	103	gram
White Rice, cooked	1/2	cup

Peas, frozen	1/4	cup
Butter*	5	gram
Garden Salad		
Iceburg Lettuce, shredded	3/4	cup
Cucumber, sliced with peel	2	slices, 1/4" each
Red Cherry Tomatoes	2	each
Baby Carrots	2	each
Shredded Cheddar Cheese, 2% Milkfat	14	gram
Ranch Dressing, fat free*	1/2	Tbsp
**Day 3**		
**Breakfast**		
Pancakes[#]	4	each
Butter*	5	gram
Syrup, No Added Sugar Log Cabin	2	Tbsp
Land O Frost Canadian Bacon Natural Hickory Smoked	49	gram
**Lunch**		
Cheesy Turkey Noodle Casserole[#]	1	serving
Pasta, corn, cooked	74	gram
**Mid-Afternoon Snack**		
Skim Milk	8	fl oz
Snackwell's Fat Free Devil's Food Cookie	3	each
**Dinner**		
Beef Tenderloin, broiled*	90	gram
Mushrooms, raw, sliced	1/2	cup
Heinz Home Style Savory Beef Gravy	2	Tbsp
Ore Ida Steam & Mash Cut Potatoes	1/2	cup
Cut Green Beans, No Salt Added	1/2	cup
King's Hawaiian Honey Wheat Roll	1	each
Butter*	5	gram
**Day 4**		
**Breakfast**		
Triple Berry Muffin*	2	each
Kroger Carbmaster Yogurt (any flavor)	1	each (170 gram)
Almonds, Blanched*	7	gram
Quaker Kretschmer Wheat Germ*	7	gram
Vanilla Drink[#]	~12	fl oz (297 gram)
Chocolate Flavoring Syrup*	10	gram
**Lunch**		
Potato Chowder[#]	1	serving
Healthy Life 100% Whole Wheat Bread	1	slice
Pimento*	6	gram
Shredded Cheddar Cheese, 2% Milkfat	28	gram
Fat Free Mayonnaise*	1	Tbsp
Baby Carrots	6	each
Diet Pepsi	1	can (12 fl oz)
**Mid-Afternoon Snack**		
Clif Builders Bar	1	serving (36 gram)
**Dinner**		
Barilla Plus Rotini Pasta, dry weight	28	gram
Prego Meatless Spaghetti Sauce	1/2	cup
Honeysuckle White Breast of Turkey, raw	83	gram
Parmesan Cheese	3	Tbsp
Caesar Salad		
Lettuce, shredded	3/4	cup
Ken's Steakhouse Light Creamy Caesar Dressing	1	Tbsp
**Day5**		
**Breakfast**		
Gravy[#]	1	serving (178 gram)

Jimmy Dean Fully Cooked Sausage Links	1	link (21 gram)
Pears, juice pack	1/2	cup
Skim Milk	8	fl oz
**Lunch**		
Pepperoni Pizza		
Pizza Crust#	1	each
Rago Pizza Sauce	1/2	cup
Pepperoni	14	gram
Mozzarella Cheese	20	gram
Garden Salad		
Shredded lettuce, iceberg	3/4	cup
Cucumber, sliced with peel	2	slices, 1/4" each
Red Cherry Tomatoes	2	each
Baby Carrots	2	each
Ranch Dressing, fat free*	1	Tbsp
Fruit Smoothie#	1	serving (250 gram)
Peaches, canned, water packed	1	cup
**Mid-Afternoon Snack**		
Granola*		
Kroger 100% Whole Grain Oats	19	gram
Slivered almonds	6	gram
Vital wheat gluten	8	gram
Log Cabin NSA pancake syrup	4	gram
Splenda	1	gram
Dried banana	5	gram
**Dinner**		
Kentucky Legend Ham	149	gram
Kroger Roasted Redskin Potatoes, frozen	1/2	cup
Olive Oil	2	tsp
Parmesan Cheese	2	Tbsp
Peas, frozen	1/2	cup
**Day 6**		
**Breakfast**		
Pancakes#	4	each
Butter*	5	gram
Syrup, No Added Sugar Log Cabin	2	Tbsp
Land O Frost Canadian Bacon Natural Hickory Smoked	49	gram
**Lunch**		
Cheesy Tuna Casserole#	1	serving
Pasta, corn, cooked	74	gram
**Mid-Afternoon Snack**		
Skim Milk	8	fl oz
Snackwell's Fat Free Devil's Food Cookie	3	each
**Dinner**		
Beef Burrito		
Ole Extreme Wellness Whole Wheat Tortilla	1	each
Tyson Grilled & Ready Beef Fajita	88	gram
Lettuce, shredded	1/2	cup
Shredded Cheddar cheese, 2% Milkfat	22	gram
Southwestern Ranch Dressing*	1	Tbsp (16 gram)
Tostitos Scoops, Baked	10	each
Salsa	1/4	cup
**Day 7**		
**Breakfast**		
Blueberry Muffin*	2	each
Kroger Carbmaster Yogurt (any flavor)	1	each (170 gram)
Almonds, Blanched*	6	gram
Quaker Kretschmer Wheat Germ*	9	gram

Vanilla Drink#	~12	fl oz (297 gram)
Chocolate Flavoring Syrup*	10	gram
**Lunch**		
Ham & Cheese Hashbrown Casserole#	1	serving
Cut Green Beans, No Added Salt	1	cup
Butter*	5	gram
**Mid-Afternoon Snack**		
Soy Nuts*	28	gram
Diet Soda	1	can (12 fl oz)
**Dinner**		
Kroger Frozen Grilled Chicken Breast, fully cooked	95	gram
Potato, small baked	1	each
Oscar Mayer Turkey Bacon	1	slice
Sour Cream, fat free	1	Tbsp
Shredded Cheddar Cheese, 2% Milkfat	14	gram
Carrots, slice, frozen	1/2	cup
Butter*	10	gram

* Indicates foods were provided to participant

# Indicates that foods that were provided contained either the maltodextrin or milk protein isolate

Supplementary Table 3. The ingredient list and gram weights for the select foods and beverages that contained milk protein isolate or maltodextrin during the intervention for a 1600 kcal menu.

Foods	Weight (g)
**Gravy[1]**	
**MPI**	
Milk protein isolate	20
Peppered Gravy Mix	21
Pepper	0.2
Water	136
Total	178
**CON**	
Polycose	16
Peppered Gravy Mix	21
Pepper	0.1
Molly McButter	3
Water	137
Total	179
**Biscuit[2]**	
**MPI**	
Milk protein isolate	17
Bisquick, Heart Smart	33
Water	50
Total	100
**CON**	
Thick It	9
Polycose	6
Heart Smart Bisquick	34
Molly McButter	2
Water	49
Total	100
**Vanilla drink[1]**	
**MPI**	
Milk protein isolate	22
Coffeemate French Vanilla FAT FREE	24
Splenda	1.5
Vanilla Powder	1
Water	249
Total	297
**CON**	
Thick-it	9
Coffeemate French Vanilla FAT FREE	36
Water	250
Total	296
**Blueberry and triple berry muffin[2]**	
**MPI**	
Milk protein isolate	15
Triple Berry Muffin Mix (Betty Crocker)	46
Water	42
Total	103
**CON**	
Thick It	7
Polycose	7
Triple Berry Muffin Mix (Betty Crocker)	46
Water	43
Total	103
**Pancake[1]**	
**MPI**	
Milk protein isolate	37

Bisquick Heart Smart	47
Egg yolk	7.5
Water	156
Total	248
**CON**	
Bisquick Heart Smart	104
Egg yolk	7.5
Water	136
Total	247
**Corn chowder[1]**	
**MPI**	
Milk protein isolate	37
Minor's Low-Sodium Vegetable Base	10
Minor's White Wine Cream Sauce Concentrate	39
Pictsweet Seasoning Blend	59
Spices, pepper, black	0.5
Corn	34
Water	220
Total	400
**CON**	
Thick It	11
Polycose	2
Minor's Low-Sodium Vegetable Base	10
Minor's White Wine Cream Sauce Concentrate	39
Pictsweet Seasoning Blend	59
Spices, pepper, black	0.5
Corn	34
Water	221
Total	400
**Potato Chowder[1]**	
**MPI**	
Milk protein isolate	37
Minor's Low-Sodium Vegetable Base	10
Minor's White Wine Cream Sauce Concentrate	39
Pictsweet Seasoning Blend	59
Spices, pepper, black	0.5
O'Brian potatoes	85
Water	220
Total	451
**CON**	
Thick It	11
Polycose	2
Minor's Low-Sodium Vegetable Base	10
Minor's White Wine Cream Sauce Concentrate	39
Pictsweet Seasoning Blend	59
Spices, pepper, black	0.5
O'Brian potatoes	85
Water	221
Total	450
**Cheesy turkey casserole[1]**	
**MPI**	
Milk protein isolate	37
Minor's Low-Sodium Vegetable Base	10
Minor's White Wine Cream Sauce Concentrate	39
Pictsweet Seasoning Blend	59
Spices, pepper, black	0.5
Pasta, corn, cooked	73
Turkey luncheon meat	25

Cheddar cheese	18
Peas	17
Water	220.
Total	426
**CON**	
Thick It	10
Polycose	26
Minor's White Wine Cream Sauce Concentrate	39
Minor's Low-Sodium Vegetable Base	10
Pictsweet Seasoning Blend	59
Spices, pepper, black	0.5
Turkey luncheon meat	19
Cheddar cheese	23
Peas	17
Water, tap	221
Total	417
**Cheesy tuna casserole[1]**	
**MPI**	
Milk protein isolate	37
Minor's Low-Sodium Vegetable Base	10
Minor's White Wine Cream Sauce Concentrate	39
Pictsweet Seasoning Blend	59
Spices, pepper, black	0.5
Canned tuna	18
Cheddar cheese	16
Peas	17
Water	220.
Total	425
**CON**	
Thick It	10
Polycose	26
Minor's White Wine Cream Sauce Concentrate	39
Minor's Low-Sodium Vegetable Base	10
Pictsweet Seasoning Blend	59
Spices, pepper, black	0.5
Canned tuna	17
Cheddar cheese	19
Peas	17
Water, tap	221
Total	417
**Pizza crust[1]**	
**MPI**	
Milk protein isolate	25
Bisquick, Heart Smart	17
Egg Yolk	3
spices	0.1
Water	21
Total	67
**CON**	
Thick It	11
Polycose	14
Bisquick, Heart Smart	17
Egg Yolk	3
spices	0.1
Water	22
Total	67
**Fruit smoothie[2]**	
**MPI**	

Milk protein isolate	12
Jamb Razzmataz Smoothie Mix	106
Trop 50 Pomegranite Blueberry Juice	108
Splenda	1
Strawberries, frozen unsweetened	23
Total	250
**CON**	
Polycose	12
Jamb Razzmataz Smoothie Mixz	106
Trop 50 Pomegrante Blueberry Juice	108
Splenda	1
Strawberries, frozen unsweetened	23
Total	
**Ham and cheese hashbrown casserole[1]**	
**MPI**	
Milk protein isolate	3
Cream of Celery Soup	68
Hashbrowns	178
Cream Cheese, light	25
Kentucky legend	17
Cheddar cheese	17
Water	78
Total	419
**CON**	
Thick It	16
Polycose	18
Cream of Celery Soup	68
Hashbrowns	178
Cream Cheese, light	25
Kentucky legend	13
Cheddar cheese	20
Water	78
Total	417

[1]These foods were manipulated based on the participants body weight

[2]These foods were kep constant regardless of participants body weight

Abbreviations: CON, control intervention group consuming a Western-style eating pattern including 0.7 g carbohydrate/kg/d from maltodextrin; MPI, intervention group consuming a Western-style eating pattern including 0.7 g protein/kg/d from milk protein isolate

Supplemental Table 4. Average daily nutrient intake over 7 days for an 88 kg female during the 3-week weight maintenance baseline period and the 16-week weight loss intervention period*

	Baseline	Intervention	
		CON	MPI
Energy (kcal)	2358 ± 7	1612 ± 13	1614 ± 13
Total fat (g) (% energy)	81 ± 3 (31 ± 1)	50 ± 1 (28 ± 0)	50 ± 1 (28 ± 1)
Saturated fat (g) (% energy)	34 ± 3 (13 ± 1)	14 ± 3 (8 ± 2)	14 ± 3 (8 ± 2)
Monounsaturated fat (g)	16 ± 2	9 ± 2	9 ± 3
Polyunsaturated fat (g)	10 ± 3	3 ± 2	3 ± 2
Total carbohydrate (g) (% energy)	344 ± 6 (58 ± 1)	225 ± 5 (56 ± 1)	167 ± 4 (41 ± 1)
Total sugars (g)	197 ± 15	29 ± 12	27 ± 14
Total fiber (g)	21 ± 3	13 ± 3	15 ± 3
Protein (g) (% energy)	70 ± 0 (12 ± 0)	70 ± 1 (17 ± 0)	131 ± 1 (33 ± 0)
Protein (g/kg)	0.8 ± 0.0	0.80 ± 0.01	1.49 ± 0.01
Total cholesterol (mg)	167 ± 45	124 ± 3	126 ± 35
Sodium (mg)	4134 ± 632	1854 ± 486	2043 ± 436
Calcium (mg)	661 ± 116	749 ± 157	1173 ± 423
Vitamin D (µg)	77 ± 48	62 ± 56	60 ± 53

*Values are mean ± SD. A 1600 kcal/d intervention diet represents the mode among participants.
